# Analysis of the threshold range of ROS concentration in winter rapeseed of the *Brassica napus* type

**DOI:** 10.3389/fpls.2025.1673768

**Published:** 2025-09-15

**Authors:** Weiliang Qi, Wancang Sun

**Affiliations:** ^1^ School of Agriculture and Bioengineering, Longdong University, Qingyang, China; ^2^ Collaborative Innovation Center for Longdong Dryland Crop Germplasm Improvement and Industrialization, Longdong University, Qingyang, China; ^3^ Gansu Dryland Research Center of Winter Wheat Germplasm Innovation and Application Engineering, Longdong University, Qingyang, China; ^4^ Gansu Collaborative Innovation of Academicians and Experts on Dryland Agriculture in the Loess Plateau, Longdong University, Qingyang, China; ^5^ Agronomy College, Gansu Agriculture University, Lanzhou, China

**Keywords:** *Brassica napus*, O_2_^-^, H2O2, seed germination, ROS - reactive oxygen species

## Abstract

The threshold range of reactive oxygen species (ROS) concentration remains a critical challenge and focal point in future research concerning its influence on the growth and development of both beneficial and harmful plants. This study demonstrates that as the concentration of hydrogen peroxide (H_2_O_2_) increases from 0.0% to 0.6%, the seed germination rate gradually rises. At 0.6% H_2_O_2_, the germination rate peaks at 94.67%, accompanied by the maximum activities of superoxide dismutase (SOD), peroxidase (POD), and catalase (CAT). However, with further increases in H_2_O_2_ concentration (0.7% – 1.3%), the seed germination rate and antioxidant enzyme activity gradually decline, while the levels of superoxide anion (O_2_^-^) and H_2_O_2_ accumulate progressively. This suggests that higher H_2_O_2_ concentrations impair the ROS scavenging capacity in cabbage-type rapeseed, leading to increased ROS production and subsequent inhibition of growth and development. At half-lethal H_2_O_2_ concentrations (1.4%–1.5%), the seed germination rate before rehydration is significantly reduced to 10.97% and 9.03%, respectively, but can recover to approximately 50% after rehydration. H_2_O_2_ concentrations exceeding 2.2% are lethal, resulting in a 0% seed germination rate both before and after rehydration; notably, the post-rehydration germination rate remains below 10%. At these concentrations, the levels of O_2_^-^, SOD, POD, and CAT decrease to their minimum values, indicating that high exogenous H_2_O_2_ concentrations induce cell death, which in turn suppresses ROS production and inactivates ROS-scavenging enzyme activity. Consequently, cellular osmotic potential increases, leading to the accumulation of high concentrations of exogenous H_2_O_2_ within cells.

## 1 Introduction

Seed germination represents the initial and pivotal step in a plant’s life cycle (Chen et al., 2000). Meanwhile, seed germination rate - serving as a core indicator of a seed’s germination capacity - acts as a key determinant of successful crop growth and directly influences final crop yields (Tania et al., 2022). Numerous research reports have demonstrated that reactive oxygen species (ROS) can induce and promote seed germination (Bailly, 2019) playing diverse roles throughout the plant growth process (Chu et al., 2022). Kranner et al. (2010) conducted experiments using pea seeds and observed high levels of hydrogen peroxide (H_2_O_2_) and low concentrations of superoxide radicals (O_2_^-^) at the early stage of imbibition. Furthermore, the elongation of the radicle following germination was found to be significantly correlated with an increase in O_2_^-^ levels. This suggests that ROS production is an early and consistent event in the imbibition and germination processes across different seed types (Liu et al., 2017). Studies have found that exogenous application of H_2_O_2_ can break the dormancy of potato tubers (Liu et al., 2017), *Arabidopsis* (Oracz et al., 2007), *Hedysarum scoparium* (Su et al., 2016) and sugarbeet (Chu et al., 2022)seeds. According to the “oxidative window” hypothesis proposed by Bailly (2019), only a critical range of ROS concentrations alleviates dormancy while levels below or above the critical range impair germination. Recent studies have further confirmed that the effects of exogenous H_2_O_2_ on promoting or inhibiting *Arabidopsis* seed germination and seedling establishment are dose-dependent (Wang et al., 2025). Specifically, low concentrations of H_2_O_2_ facilitate *Arabidopsis* seed germination, whereas higher concentrations lead to a significant suppression of germination rate (Zhou et al., 2018; Wang et al., 2025), and this process is accompanied by changes in the activities of peroxidase (POD) and superoxide dismutase (SOD) (Jiang et al., 2012; Zheng et al., 2018). In summary, these findings collectively demonstrate the significant regulatory role of exogenous H_2_O_2_ in both seed germination and early seedling growth. Accordingly, growth regulators containing H_2_O_2_ can be applied through various methods, including seed soaking prior to sowing, incorporation into the growth medium, or foliar spraying on young plants, which can enhance both agricultural productivity and economic returns. It is widely accepted that only H_2_O_2_ within an appropriate range can promote seed germination. Both excessively low and high H_2_O_2_ concentrations are detrimental to germination, with high concentrations inhibiting plant growth and triggering programmed cell death. A key question then arises: what is the threshold range within which H_2_O_2_ act as signaling molecules to participate in plant growth and development? This remains a prominent and challenging issue in ROS research.

Currently, research on the ROS threshold in cruciferous rapeseed varieties remains relatively limited. Accordingly, this study employs 16NTS309, a cold-resistant rapeseed variety bred by our research group, as the research subject. *B. napus* of 16NTS309 having a strong cold tolerance, which could over winter in the 36°73′N area at an altitude of 1,517 m. These are the essential *B. napus* germplasm resources having strong cold tolerance levels used for breeding in northern China. The present study examined the effects of different concentrations of H_2_O_2_ (a type of ROS) on rapeseed, including its germination rate and the contents of ROS (O_2_^-^ and H_2_O_2_) as well as antioxidant enzymes (SOD, POD, and CAT). The objectives were to clarify the threshold range at which ROS promote *B.napus* growth and to verify the impact of high H_2_O_2_ concentrations on rapeseed growth and development. This study furnishes valuable theoretical guidance for the application of H_2_O_2_ in agricultural production, thereby facilitating yield improvement in rapeseed varieties. Moreover, it yields practical reference value for relevant agricultural practices and production management.

## 2 Experimental materials and treatment methods

### 2.1 Experimental materials

Mature and plump seeds of *B.napus* winter rapeseed cultivar 16NTS309 were selected, thoroughly washed, air-dried, and vernalized at a low temperature of 4 °C for 12 hours to break seed dormancy.

### 2.2 Experimental treatments

#### 2.2.1 Exploration of ROS threshold range

In this experiment, 31 different concentrations of H_2_O_2_ treatments were established to investigate the effects of varying H_2_O_2_ levels on the germination rate, plant growth length, and intracellular ROS, including O_2_^-^ and H_2_O_2_, in *B.napus* seeds. The objective was to determine the threshold range within which ROS either promotes or inhibits plant growth.

Initially, a 30% H_2_O_2_ solution was used as the stock solution, and various dilutions were prepared according to the formula C1V1 = C2V2 (30% * X = C2 * 100). The 31 gradient concentrations included: 0.0% (ddH_2_O control), 0.1%, 0.2%, 0.3%, 0.4%, 0.5%, 0.6%, 0.7%, 0.8%, 0.9%, 1%, 1.1%, 1.2%, 1.3%, 1.4%, 1.5%, 1.6%, 1.7%, 1.8%, 1.9%, 2%, 2.1%, 2.2%, 2.3%, 2.4%, 2.5%, 2.6%, 2.7%, 2.8%, 2.9%, and 3%. Each treatment involved 1000 seeds, evenly distributed into 100 culture bottles with 10 seeds per bottle, and the experiment was conducted in triplicate.

Subsequently, the seeds were rinsed three times with distilled water and vernalized at 4 °C for 24 hours. Culture bottles were sterilized, dried, and lined with a single layer of sterile filter paper. Each bottle was then treated with the corresponding concentration of H_2_O_2_. Seeds were evenly placed in the bottles and cultivated in a controlled growth chamber maintained at 25°C with a light intensity of 3000 Lx under a 16-hour photoperiod. Due to the volatility of H_2_O_2_, which may affect the accuracy of the results, we have strictly controlled the experimental conditions through the following operations: 1)Every 24 hours, leave the culture bottles open for 3 hours, and use filter paper to absorb the excess H_2_O_2_ liquid at the bottom of the bottles, then add a small amount of freshly prepared H_2_O_2_ of each concentration gradient; 2) Immediately cover the bottles after each H_2_O_2_ replacement to ensure the airtightness of the culture environment. All these operations are to ensure the reliability of the experimental data.

This procedure was repeated daily for 4d, during which germination rates and growth trends were recorded. On the 4d, intracellular levels of H_2_O_2_, O_2_^-^, SOD, POD, and CAT were measured.

#### 2.2.2 Validation experiment of ROS lethal concentration and semi-lethal concentration

##### 2.2.2.1 Seed rehydration experiment

For each H_2_O_2_ treatment gradient with a germination rate below 50%, a rehydration experiment was conducted to assess the recovery of seed germination following rehydration and to determine the threshold concentrations representing the lethal and semi-lethal levels of H_2_O_2_. Initially, 21 H_2_O_2_ gradients ranging from 1% to 3% were repeated. Each treatment involved 1000 seeds, which were evenly distributed into 100 culture bottles (10 seeds per bottle). Following 4d of cultivation, the germination rates for each H_2_O_2_ concentration were recorded. Subsequently, seeds treated with 1%, 1.1%, 1.2%, 1.3%, 1.4%, 1.5%, 1.6%, 1.7%, 1.8%, 1.9%, 2%, 2.1%, 2.2%, 2.3%, 2.4%, 2.5%, 2.6%, 2.7%, 2.8%, 2.9%, and 3% H_2_O_2_ were carefully rinsed five times with distilled water. The culture bottles were also cleaned and dried. Subsequently, the seeds from each treatment were evenly placed back into the respective culture bottles, and an equal volume of ddH_2_O was added. Cultivation was continued under controlled conditions at 25 °C with a light intensity of 3000 Lx and a photoperiod of 16 hours per day for an additional 4d. Daily observations of germination rates were recorded throughout this period.

Note: If the germination rate increased significantly after rehydration and exceeded 60%, it indicated that the high H_2_O_2_ concentration had temporarily inhibited germination. If the germination rate remained around 50% after rehydration, the corresponding H_2_O_2_ concentration was considered the semi-lethal concentration for *B.napus* growth and development. If the germination rate remained below 10% after rehydration, the H_2_O_2_ concentration was classified as the lethal concentration for *B.napus*.

##### 2.2.2.2 Verification experiment

To further validate the reliability of the experimental data, normal *B.napus* seedlings were sprayed with H_2_O_2_ solutions at concentrations ranging from 1% to 3% to observe their growth responses. Initially, healthy seedlings were cultivated in culture bottles. When the plants reached a height of approximately 7 cm, they were treated by spraying with 1%, 1.2%, 1.3%, 1.4%, 1.5%, 1.6%, 1.7%, 1.8%, 1.9%, 2%, 2.1%, 2.2%, 2.3%, 2.4%, 2.5%, 2.6%, 2.7%, 2.8%, 2.9%, and 3% H_2_O_2_ solutions, respectively. Each treatment consisted of 30 culture bottles, with 10 seedlings per bottle. The treatment was continued for 4d, and the growth status of the rapeseed seedlings was observed and recorded on the fourth day.

### 2.3 Index measurement

#### 2.3.1 Seed germination

Daily observations were conducted to record germination parameters, including germination rate and seedling length. The germination rate was calculated using the following formula:


Germination rate (%)=(number of germinated seeds/total number of tested seeds)×100%.

#### 2.3.2 Qualitative detection of H_2_O_2_ and O_2_^-^

Seedlings were placed in the test tubes and immersed in 3, 3'-Diaminobenzidine (DAB) and Nitrotetrazolium blue chloride (NBT) staining solution to detect H_2_O_2_ and O_2_^−^ as described by (Wang et al., 2025). Plants were immersed for 8 h with DAB and NBT staining solution that solution should be placed away from light. After infiltration, the stained plants were bleached in an acetic acid:glycerol: ethanol (1:1:3, v/v/v) solution at 100 °C for 10 - 20min, then stored in 95% (v/v) ethanol until scanned. The experiment was repeated six times.

#### 2.3.3 Quantitative detection of SOD, POD, CAT, H_2_O_2_, and O_2_^-^

Following four days of treatment, the levels of H_2_O_2_ and O_2_^-^, as well as the enzymatic activities of SOD, POD, and CAT, were quantitatively measured in *B.napus* seedlings exposed to each H_2_O_2_ concentration gradient. POD, SOD and CAT activities were measured as described by (Qi et al., 2020).

### 2.4 Data analysis

The data were analyzed using SPSS 19.0 software, with one-way analysis of variance (ANOVA) applied to identify significant differences between the two treatment groups (*P<* 0.05). Graphical illustrations were prepared using Adobe Photoshop CC 2018 (Adobe Inc., San Jose, CA, USA).

## 3 Results

### 3.1 Effects of exogenous H_2_O_2_ on rapeseed germination rate exogenous

**Figure 1:**
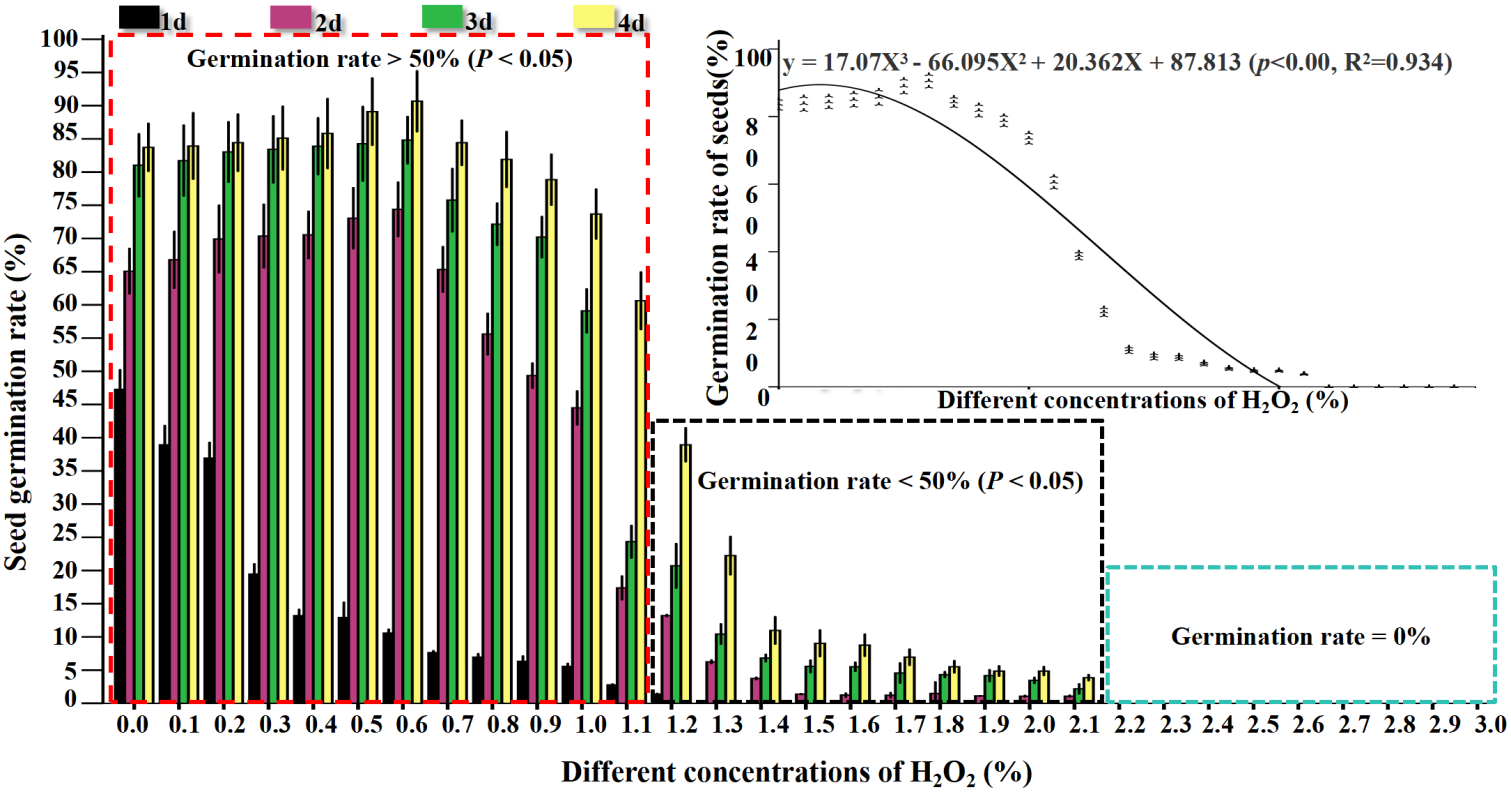
Effect of seed germination rate with different concentrations of H_2_O_2_ on *B napus*.

**Figure 2:**
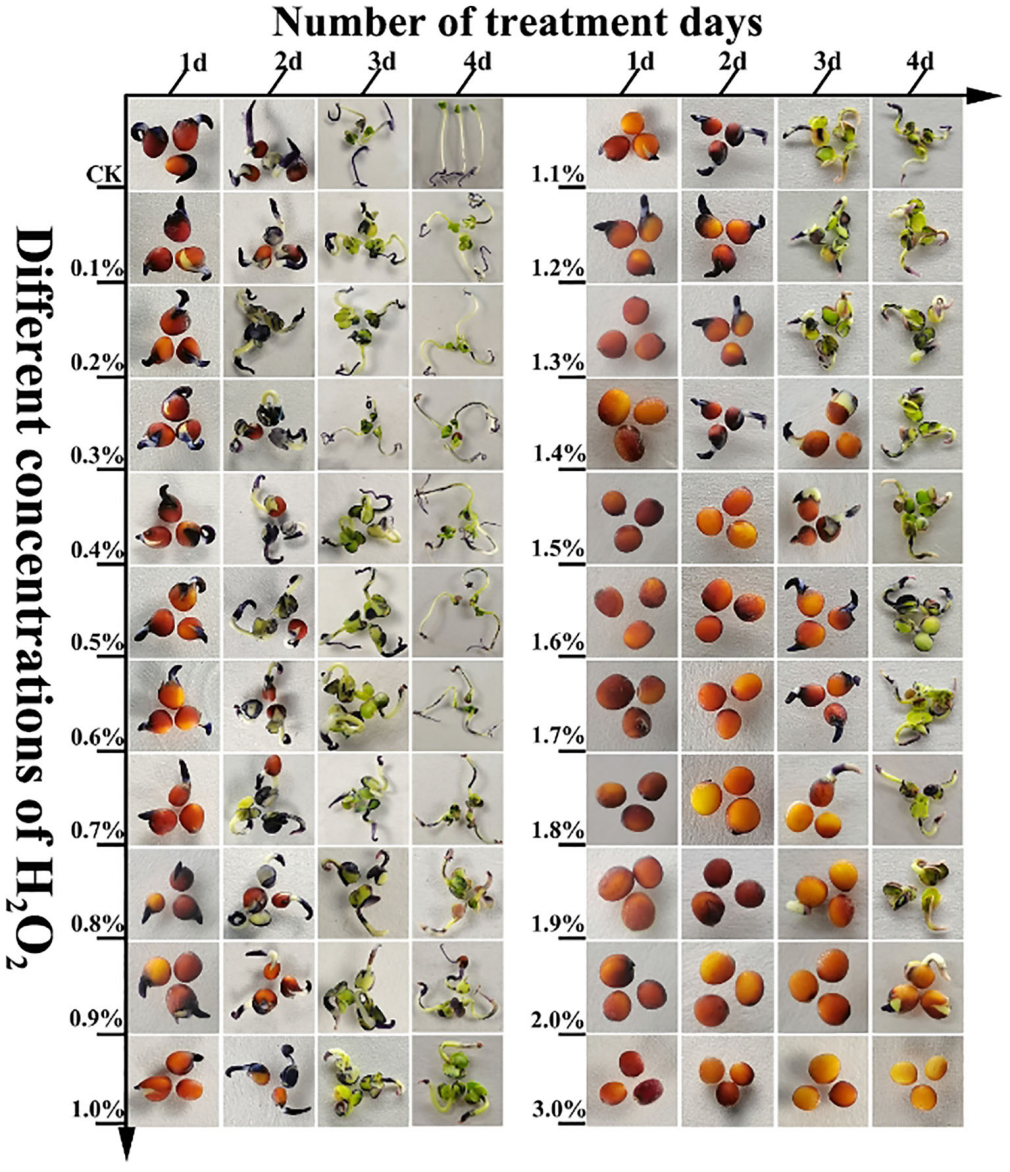
The seeds of *B.napus* 16NTS309 were treated with H_2_O_2_ and then stained with NBT to observe the distribution of O_2_^-^ in the hypocotyl, cotyledon and embryo.

**Figure 3:**
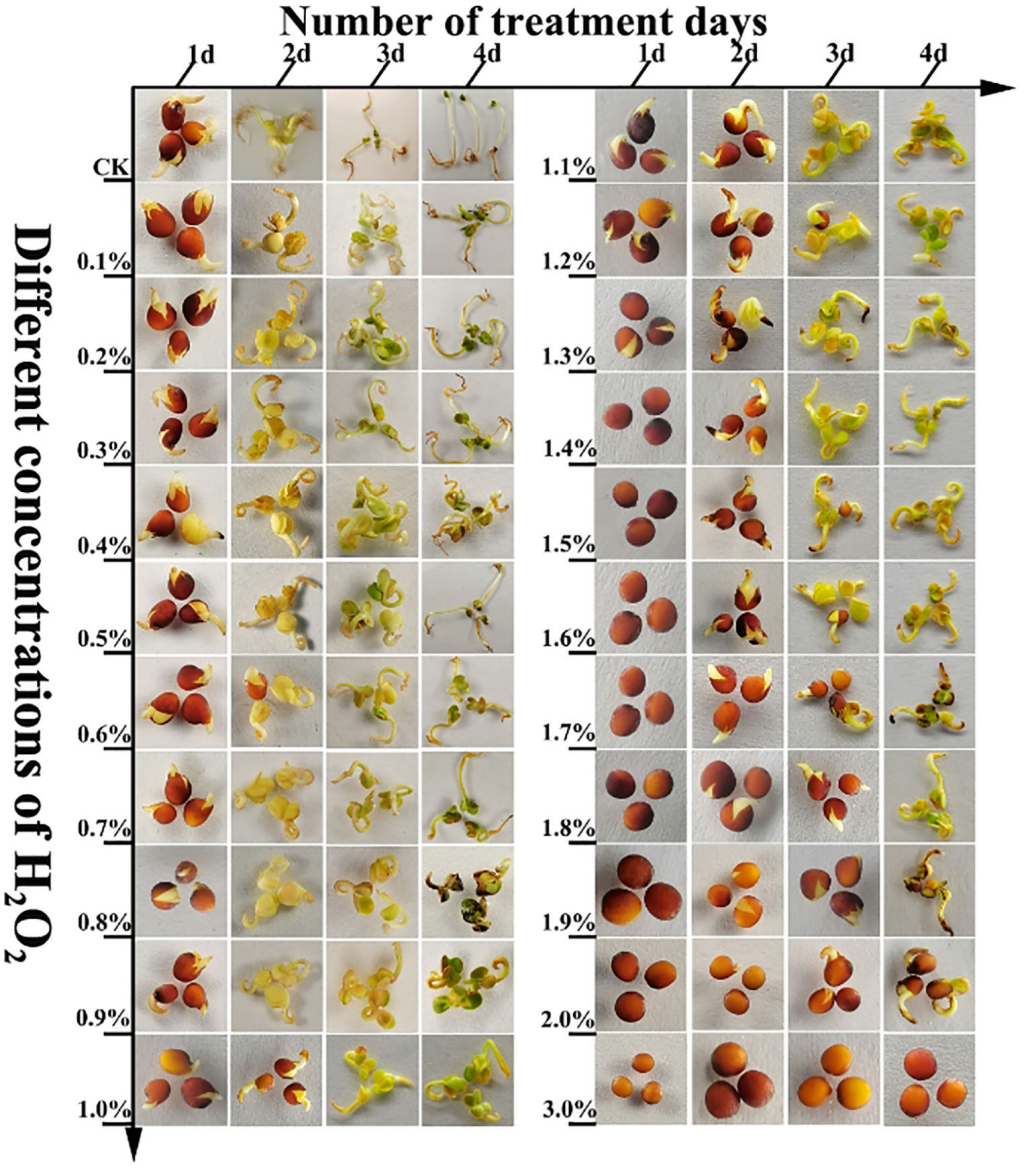
The seeds of *B.napus* 16NTS309 were treated with H_2_O_2_ and then stained with DAB to observe the distribution of H_2_O_2_ in the hypocotyl, cotyledon and embryo.

H_2_O_2_ at different concentrations exerted distinct effects on the viability of rapeseed seeds, with variations in germination rate observed across different treatment durations, as detailed below: After 1 day of treatment, the germination rate of seeds subjected to 0.0% (CK) to 3% H_2_O_2_ treatments exhibited a downward trend (**Figure 1**; **Appendix Table 1**). Among these, seeds treated with 0.0% (CK) to 1.2% H_2_O_2_ sequentially underwent swelling, embryo breakthrough of the seed coat, and accumulation of substantial O_2_^-^ at the radicle tip (**Figure 2**), with relatively low H_2_O_2_ accumulation (**Figure 3**). Specifically, the germination rate of the 0.0% (CK) treatment reached 47.22%, which was significantly higher (*P*< 0.5) than those of the 0.1% to 3% H_2_O_2_ treatments. The germination rates of the 0.1% and 0.2% H_2_O_2_ treatments followed, at 38.89% and 36.72%, respectively. For the 0.3% to 1.2% H_2_O_2_ treatments, germination rates ranged from 19.44% to 1.39%, while no germination was observed in the 1.3% to 3% H_2_O_2_ treatments.

After 2 days of treatment, the germination rate of seeds across the 0.0% (CK) to 3% H_2_O_2_ treatments displayed a trend of first increasing and then decreasing (**Figure 1**; **Appendix Table 1**). Specifically, germination rates rose in the 0.0% (CK) to 0.6% H_2_O_2_ range, peaking at 74.31% in the 0.6% H_2_O_2_ treatment (**Appendix Table 1**), indicating that an appropriate concentration of H_2_O_2_ promotes rapeseed germination. In contrast, germination rates declined in the 0.7% to 2.1% H_2_O_2_ treatments, ranging from 65.28% to 1.08%, and no germination occurred at H_2_O_2_ concentrations ≥ 2.2%, suggesting that higher H_2_O_2_ concentrations inhibit seed germination. NBT (**Figure 2**) and DAB(**Figure 3**) staining results revealed increased accumulation of O_2_^-^ in the radicle tips and cotyledon regions, with relatively low H_2_O_2_ accumulation (**Figure 3**).

After 3 days of treatment, compared with the 2nd day, the germination rate in each treatment gradient showed the most significant increase; however, seeds treated with 2.2% to 3% H_2_O_2_ still failed to germinate. Germination rates in the 0.0% (CK) to 0.9% H_2_O_2_ treatments ranged from 70.41% to 84.72%, with the highest rate (84.72%) observed at 0.6% H_2_O_2_. Seeds treated with 1% to 2.1% H_2_O_2_ exhibited varying degrees of swelling and radicle breakthrough of the seed coat, with germination rates ranging from 2.17% to 59.03% (**Figure 1**; **Appendix Table 1**).

On the 4th day, the germination rate across the 0.0% (CK) to 3% H_2_O_2_ treatments maintained the trend of first increasing and then decreasing, reaching a maximum of 94.67% at 0.6% H_2_O_2_. However, the rate of increase in germination slowed compared to the 3rd day, and no germination was still observed in the 2.2% to 3% H_2_O_2_ treatments (**Figure 1**; **Appendix Table 1**). These results confirm that an appropriate concentration of exogenous H_2_O_2_ facilitates seed germination, while both lower and higher concentrations exert inhibitory effects. Under the 0.5% and 0.6% H_2_O_2_ treatments, the germination rate of rapeseed ranged from 89.01% to 94.67%, indicating that this concentration was the most favorable for the germination of rapeseed (**Figure 1**; **Appendix Table 1**). At 1% and 1.1% H_2_O_2_, germination rates were 73.61% and 60.56%, respectively, significantly lower than that of the 0.0% (CK) treatment, confirming that higher concentrations hinder germination. At 1.2% H_2_O_2_, the germination rate was 38.89%, which is speculated to be the semi-lethal concentration for cabbage-type rapeseed seeds. At H_2_O_2_ concentrations > 1.5%, germination rates dropped below 10%, potentially representing the lethal concentration for these seeds (**Figure 1**; **Appendix Table 1**).

The curve fitting yielded the following cubic polynomial equation: y = 17.07X³ - 66.095X² + 20.362X + 87.813 (*p*< 0.001, R² = 0.934). Statistical validation of this model demonstrates two key findings: The p-value is less than 0.001, which confirms the extremely high statistical significance of the overall regression relationship, indicating that the association between the independent variable X and dependent variable y is not by chance. The coefficient of determination R² = 0.934 means the model accounts for approximately 93.4% of the total variation in the dependent variable y, reflecting excellent goodness of fit.

### 3.2 Effects of exogenous H_2_O_2_ on H_2_O_2_ and O_2_^-^ contents in *B.napus* seeds

In this study, *B.napus* seeds of 16NTS309 were continuously treated with 31 gradient concentrations of exogenous H_2_O_2_ (0.0%–3%) for 4 days. After treatment, NBT staining (for O_2_^-^, **Figure 2**) and DAB staining (for H_2_O_2_, **Figure 3**) were performed on the seeds or seedlings to investigate the variation patterns of H_2_O_2_ and O_2_^-^ contents. Staining results showed that germinating seeds accumulated abundant blue precipitates (indicating O_2_^-^) at the radicle tip, with relatively few brown precipitates (indicating H_2_O_2_). This suggests that a large amount of O_2_^-^ accumulates at the germinating tip (**Figure 3**), which is beneficial for cell division in this region. During the later stages of seed growth and development, O_2_^-^ and H_2_O_2_ were also detected in cotyledons and hypocotyls, further confirming that H_2_O_2_ and O_2_^-^ play crucial roles in seed growth and development.

However, with increasing concentrations of exogenous H_2_O_2_ (1.7%–2.1%), the accumulation of blue precipitates (O_2_^-^) decreased (**Figure 2**), while brown precipitates (H_2_O_2_) darkened progressively (**Figure 3**). This indicates that high concentrations of exogenous H_2_O_2_ inhibit the reactive oxygen species (ROS) generation mechanism, leading to reduced O_2_^-^ accumulation and increased H_2_O_2_ levels. The elevated H_2_O_2_ content may be attributed to enhanced membrane permeability caused by high exogenous H_2_O_2_ concentrations, which allows external H_2_O_2_ to penetrate into cells—likely the primary reason for the dark brown staining observed in DAB assays.

### 3.3 Quantitative analysis of H_2_O_2_ and O_2_^-^ contents

**Figure 4:**
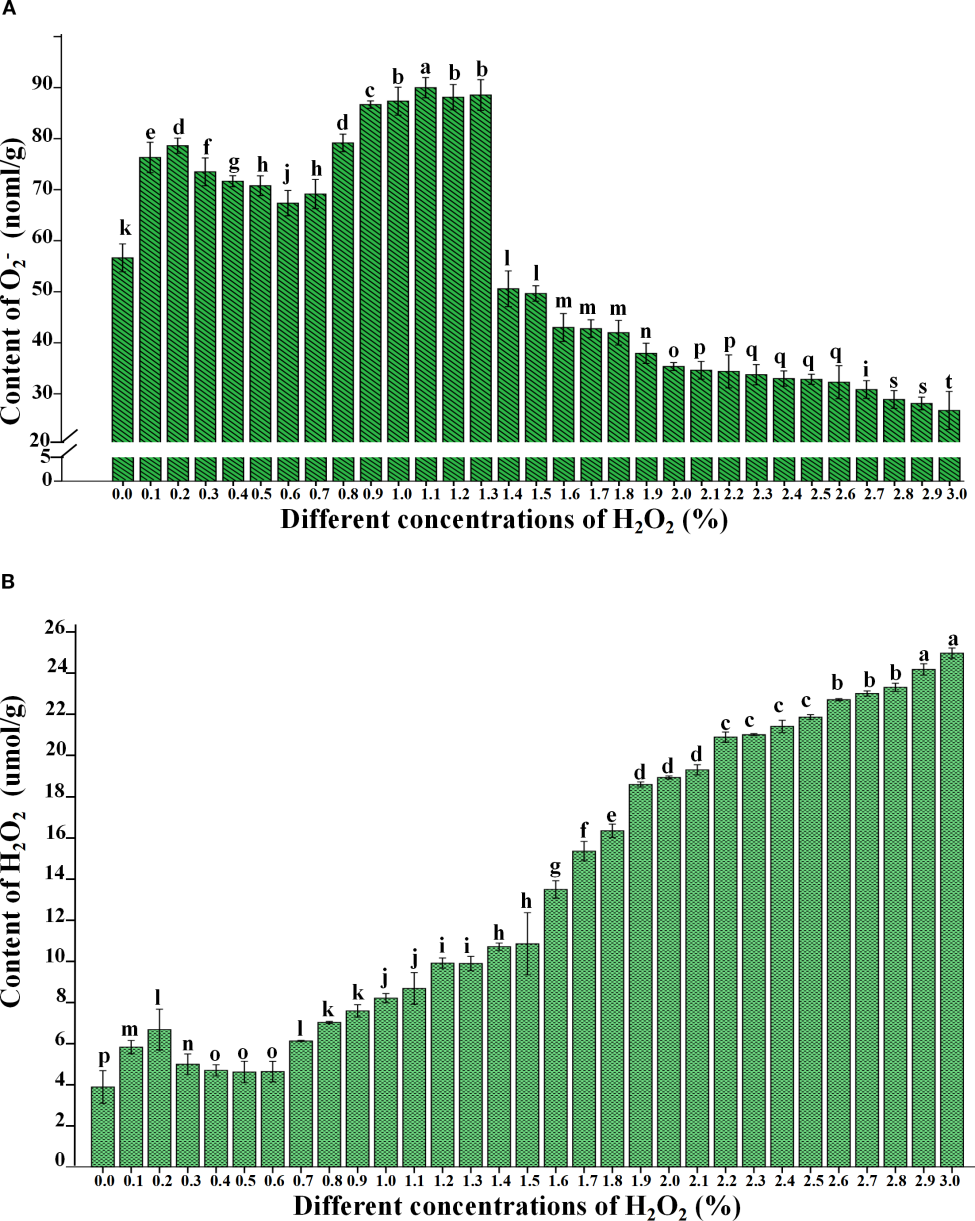
Change of O_2_^-^ **(A)** and H_2_O_2_ **(B)** content in *B.napus* seed with treated by different concentrations of H_2_O_2_. Lowercase letters (i.e., a, b, c, etc.) are used to denote the results of differential analysis, where distinct letters indicate statistically significant differences at the P < 0.05 level.

To further validate the reliability of the qualitative test results for H_2_O_2_ and O_2_^-^, this study quantitatively analyzed the contents of H_2_O_2_ and O_2_^-^ in 31 samples under each H_2_O_2_ concentration (0.0% – 3.0%) after 4 days of treatment. The results indicated that as the H_2_O_2_ concentration increased, the O_2_^-^ (**Figure 4A**) content exhibited a fluctuating trend, initially increasing, then decreasing, followed by a second increase and a final decrease (**Figure 4**; **Appendix Table 2**). In contrast, H_2_O_2_ (**Figure 4B**) content displayed a trend of initial increase, subsequent decrease, and a second increase. Significant differences were observed in both O_2_^-^ (**Figure 4A**) and H_2_O_2_ (**Figure 4B**) contents across different treatments (*P<* 0.05), indicating that varying H_2_O_2_ concentrations exerted significant effects on plant growth and germination.

Compared with the control treatment (0.0% H_2_O_2_), the O_2_^−^ and H_2_O_2_ (**Figure 4**; **Appendix Table 2**) contents in seeds treated with 0.1% and 0.2% H_2_O_2_ showed an increasing trend and were significantly higher than those in the control group. Specifically, the O_2_^−^ contents were 76.32 nmol/g and 78.62 nmol/g, while the H_2_O_2_ contents were 5.82 μmol/g and 6.67 μmol/g, respectively. These findings suggest that low concentrations of exogenous H_2_O_2_ can stimulate the intracellular accumulation of ROS, including O_2_^−^ and H_2_O_2_. However, higher concentrations of ROS did not show a significant promoting effect on rapeseed germination. Moreover, the O_2_^−^ and H_2_O_2_ contents in seeds treated with 0.3%–0.6% H_2_O_2_ were significantly higher than in the control group but markedly lower than those in seeds treated with lower (0.1%–0.2%) and higher (0.8%–1.3%) H_2_O_2_ concentrations. This suggests that both low and high concentrations of H_2_O_2_ can induce greater ROS production within seeds, which may partially inhibit germination.

Upon treatment with 1.4% H_2_O_2_, the O_2_^−^ content in seeds exhibited a sharp decline (**Figure 4A**; **Appendix Table 2**), whereas the H_2_O_2_ content showed a rapid increase. This observation was consistent with the NBT (**Figure 2**) and DAB (**Figure 3**) staining results. A plausible explanation is that high H_2_O_2_ concentrations may lead to seed mortality, thereby severely impairing the cellular capacity to produce and scavenge ROS. Despite this, the ongoing processes of absorption and imbibition within the non-viable seeds allowed for the accumulation of exogenous H_2_O_2_, resulting in a sharp rise in H_2_O_2_ levels between 1.9% and 3.0%. This result aligns with the DAB staining data.

### 3.4 Effects of exogenous H_2_O_2_ on endogenous SOD, POD, and CAT activities in *B.napus* seeds

**Figure 5:**
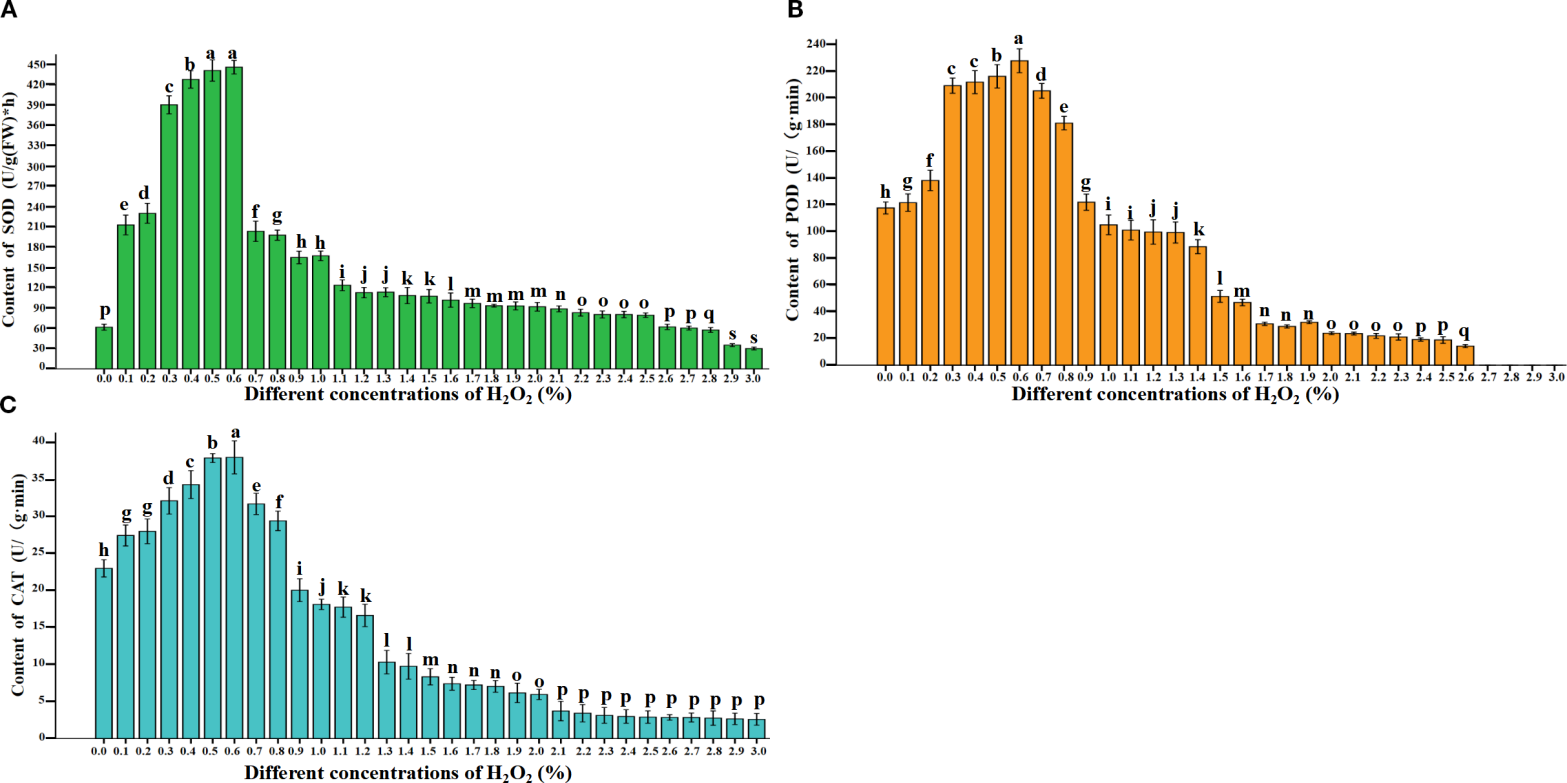
Effects of exogenous H_2_O_2_ concentration treatments on SOD **(A)**, peroxidase POD, **(B)**, and catalase CAT, **(C)** in rapeseed seedlings. Lowercase letters (i.e., a, b, c, etc.) are used to denote the results of differential analysis, where distinct letters indicate statistically significant differences at the P < 0.05 level.

This study determined the activities of SOD, POD, and CAT in 31 samples after 4 days of treatment with exogenous H_2_O_2_ at concentrations ranging from 0.0% to 3%. The results showed that as the H_2_O_2_ concentration increased, the activities of SOD (**Figure 5A**), POD (**Figure 5B**), and CAT (**Figure 5C**) exhibited a consistent trend of initial increase followed by decrease. Statistical analysis revealed significant differences in enzyme activities among all treatments (*P*<0.05), indicating that varying H_2_O_2_ concentrations significantly affect the dynamics of SOD, POD, and CAT activities during seed germination (**Figure 5**; **Appendix Table 2**). Notably, SOD, POD, and CAT activities were significantly higher in seeds treated with 0.3% – 0.6% H_2_O_2_ compared to other treatments. This finding aligns with the earlier observation that 0.3%–0.6% H_2_O_2_ is optimal for seed germination, suggesting that these enzymes play a critical role in maintaining ROS (H_2_O_2_ and O_2_^-^) at physiologically appropriate levels-likely explaining the lower intracellular ROS content observed under these concentrations. However, compared with the normal treatment (0.0%), the activities of SOD (**Figure 5A**), POD (**Figure 5B**), and CAT (**Figure 5C**) enzymes were significantly enhanced by low concentrations of H_2_O_2_ (0.1% and 0.2%). Nevertheless, these enzyme activities were markedly reduced at higher H_2_O_2_ concentrations (0.3%–0.6%). When the H_2_O_2_ concentration exceeded 0.7%, increased H_2_O_2_ levels progressively inhibited seed germination as well as the activities of SOD (**Figure 5A**), POD (**Figure 5B**), and CAT (**Figure 5C**) enzymes. This suggests that elevated H_2_O_2_ concentrations are detrimental to seed germination, as they suppress antioxidant enzyme activities, thereby impairing the capacity to scavenge ROS and ultimately leading to increased seed mortality.

### 3.5 Verification experiment for ROS threshold

Previous results indicated that as exogenous H_2_O_2_ concentration increased (1% – 3%), seed growth and development were significantly inhibited. After treatment with 1.2% H_2_O_2_, the seed germination rate was 35.6%, from which it can be inferred that this concentration represents the half-lethal threshold. In contrast, following treatment with 1.5% H_2_O_2_, the seed germination rate dropped to 9.5%, thereby indicating that this concentration is the lethal concentration of H_2_O_2_. To verify this experimental result, in this study, the seeds that had been treated with H_2_O_2_ for 4 days were subjected to H_2_O treatment again.

**Figure 6:**
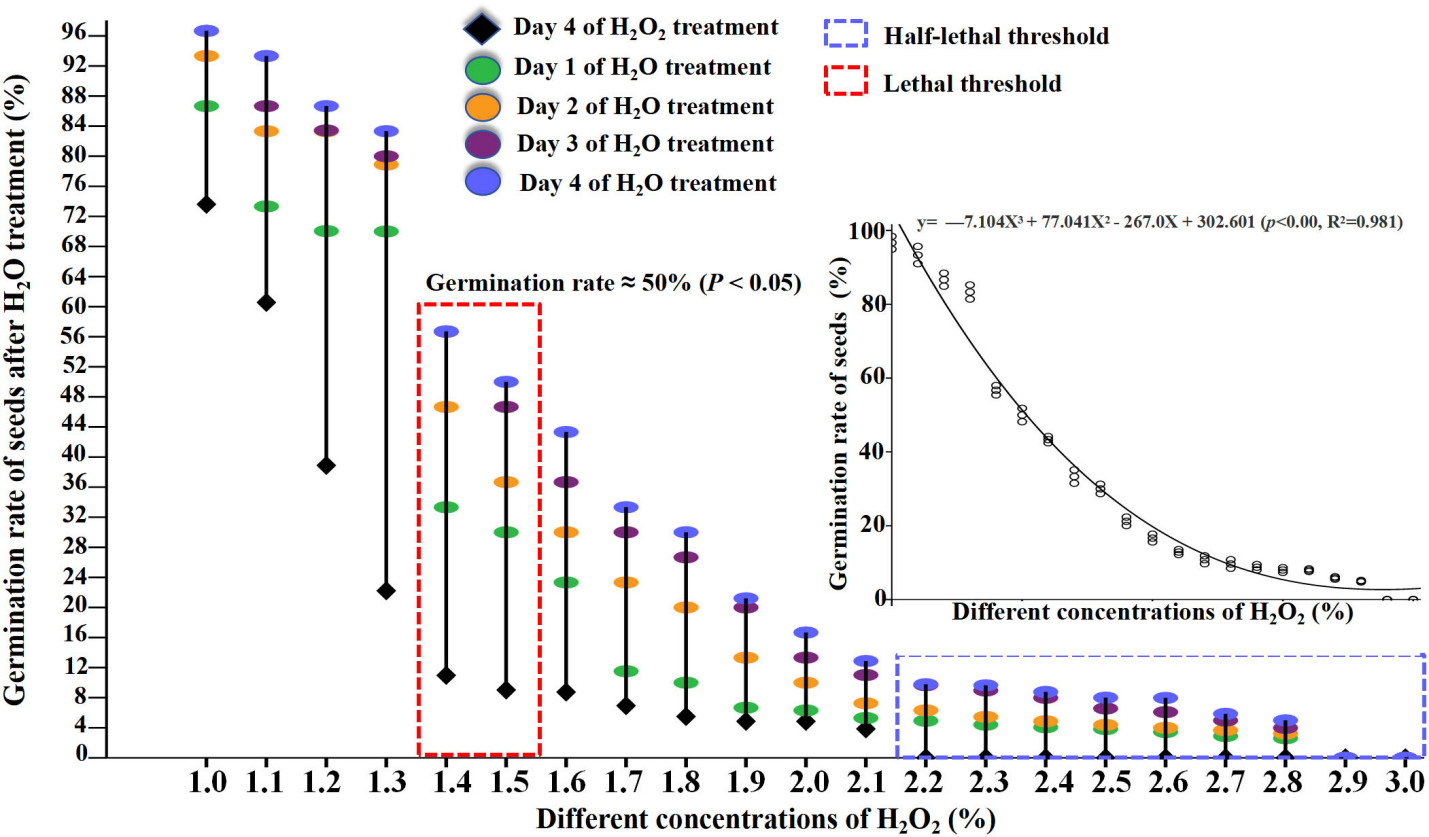
After the seeds were pretreatment with H_2_O_2_ (1%-3%) for 4d and then re-watering (4d), effect of H_2_O_2_ and re-watering on seed germination rate of *B.napus* were counted.

The results revealed significant changes in germination rates across all H_2_O_2_ treatments after rehydration, compared to pre-rehydration levels (**Figure 6**; **Appendix Table 3**). Specifically: Seeds treated with 1%–1.3% H_2_O_2_ exhibited germination rates of 93.3%, 83.3%, 82.2%, and 80.0% after rehydration, with particularly notable increases in the 1.2% and 1.3% groups (**Figure 6**; **Appendix Table 3**). This suggests that while high H_2_O_2_ concentrations initially inhibit germination, rehydration can alleviate this inhibition, thereby increasing germination rates. Consequently, 1.2% H_2_O_2_ is not within the semi-lethal concentration range.

**Figure 7:**
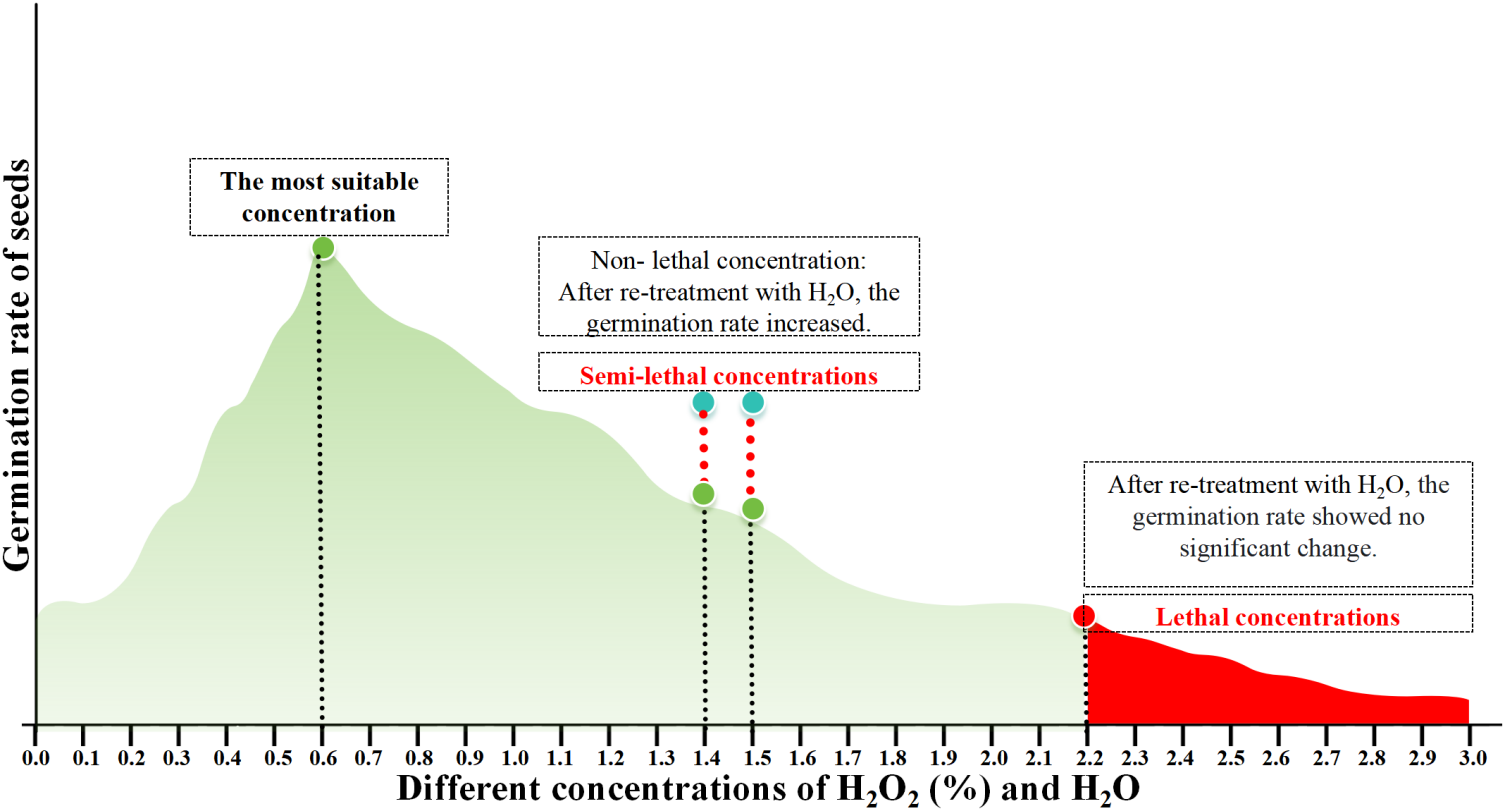
H_2_O_2_ treatment mode diagram.

After 4 days of supplementary H_2_O treatment, the germination rates of seeds previously treated with 1.4% and 1.5% H_2_O_2_ increased to 56.72% and 50%, respectively (**Figure 6**; **Appendix Table 3**). This indicates that 1.4% and 1.5% H_2_O_2_ may instead represent the semi-lethal concentrations for the germination of cabbage-type rapeseed. While those treated with 2.2%–3% H_2_O_2_ maintained germination rates below 10% (approaching 0 with increasing concentration). These results indicate that H_2_O_2_ concentrations exceeding 2.2% are likely lethal for cabbage-type rapeseed germination (**Figure 7**).

**Figure 8:**
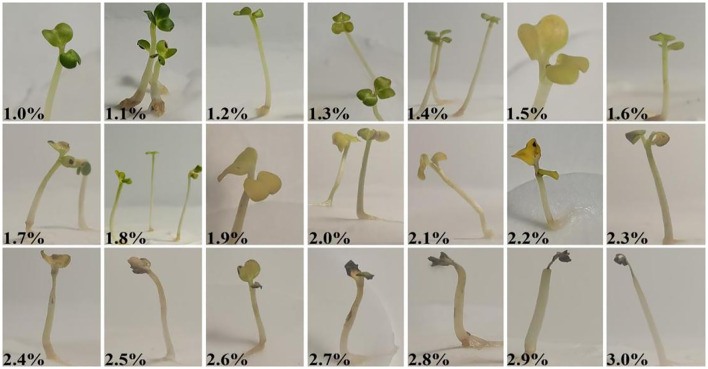
The normal growth of *B. napus* 16NTS309 seedlings were treated by spraying high concentration H_2_O_2_ (1.0%-3.0%) to verify the effect of high concentration H_2_O_2_ on the growth and development of 16NTS309 seedlings.

To further confirm these findings, an additional experiment was conducted: normal-growing cabbage-type rapeseed plants were sprayed with high-concentration H_2_O_2_ (1.0%–3.0%). Observations showed that leaf yellowing intensified with increasing H_2_O_2_ concentration, and extensive leaf necrosis occurred at concentrations >2.2% (**Figure 8**). These results, coupled with the inverse correlation between chlorophyll content and H_2_O_2_ concentration, further validate that high levels of ROS (specifically H_2_O_2_) exert inhibitory or lethal effects on the growth and development of cabbage-type rapeseed.

The cubic polynomial equation derived from curve fitting is: y = -7.104X³ + 77.041X² - 267.0X + 302.601 (*p*< 0.001, R² = 0.981). Statistical indicators confirm the model’s excellent fitting performance, as elaborated below: Statistical significance (p< 0.001):This indicates the overall regression relationship of the cubic polynomial is statistically highly significant. It effectively rules out the possibility that the association between the independent variable (X) and dependent variable (y) is driven by random errors. Goodness of fit (R² = 0.981):The coefficient of determination (R²) reaches 0.981, meaning the model accounts for approximately 98.1% of the total variation in the dependent variable (y). This reflects the model’s strong explanatory power for the data, high fitting accuracy, and its ability to well capture the nonlinear relationship between X and y.

## 4 Discussion

### 4.1 Appropriate H_2_O_2_ concentration promotes the germination of *B.napus* seeds

Studies have indicated that the germination of seeds is closely associated with the production of ROS (Zheng et al., 2009). A growing body of research by scholars has confirmed that ROS can act as signaling molecules to facilitate seed germination, including soybeans (Li et al., 2023), beans (Pereira et al., 2017), cotton (Wang et al., 2022), peas (Barba-Espín et al., 2012b), wheat (Momeni et al., 2023), and cucumbers (Ahmad et al., 2021). Nevertheless, exogenous H_2_O_2_ at different concentrations exerts distinct effects on crop growth and development: low concentrations promote seed germination, while high concentrations inhibit it (Wang et al., 2025). Chuesaard et al (2023) demonstrated that a low concentration of H_2_O_2_ (25 mM) can accelerate the germination of *Oryza sativa* seeds. Similarly, when wheat seeds were treated with H_2_O_2_ at different concentrations (0.5%, 1%, and 3%), the low concentration 0.5%H_2_O_2_ significantly increased the germination percentage. Li et al. (2014) treated *Pinus bungeana* seeds with gradient concentrations of H_2_O_2_ and recorded the highest germination rate (76.0%) at a 0.6%H_2_O_2_ concentration. In the present study, 31 H_2_O_2_ concentration gradients (ranging from 0.0% to 3.0%) were tested to assess their impact on *B.napus* seed germination. The results revealed that: with H_2_O_2_ concentration increased, the germination rate first rose and then declined. Lower concentrations of H_2_O_2_ (0.3%-0.6%)promote seed germination, resulting in a germination rate exceeding 85.303%, which is significantly higher than that of the control group (0.0%). notably, the maximum germination rate (94.67%) was achieved at 0.6% H_2_O_2_. Our research findings are consistent with Li et al (2014); Wang et al (2025) and Chuesaard et al (2023) studies. These findings indicate that a 0.6% H_2_O_2_ concentration is conducive to *B.napus* seed germination.

### 4.2 The influence of higher concentrations of H_2_O_2_ on the growth and development of *B.napus* seeds

The dual role of H_2_O_2_ exerts a marked dual effect: at low concentrations, it influences seed germination and seedling growth, whereas at higher concentrations, it induces seed death (Jeevan et al., 2015), with such impacts exhibiting a dosage-dependent pattern. In light of this characteristic, Wang et al (2025) conducted an analysis on the influence of varying concentrations of H_2_O_2_ on *Arabidopsis* seed germination, the results revealed that 2 mM H_2_O_2_ serves as a critical threshold: when this concentration is exceeded, both *Arabidopsis* seed germination and seedling growth exhibit a gradual inhibition. Chuesaard et al. (2023) demonstrated that higher concentrations H_2_O_2_(100–200 mM) exert a delaying effect on the process. Similar concentration-dependent effects have been observed across various plant species. For instance, in soybean germination experiments, exogenous application of H_2_O_2_ at 10 mM or 20 mM was found to inhibit germination (Barba-Espín et al., 2012b). Arooj et al (2024) found that higher concentrations H_2_O_2_ (1% and 3%) significantly impaired germination. After treating *B.napus* seeds with 31 distinct concentrations of H_2_O_2_, we observed comparable results: 0.6% H_2_O_2_ emerged as a critical threshold. As the concentration increased to the range of 0.7%–3.0%, the seed germination rate exhibited a declining trend, with germination completely ceasing (germination rate = 0) once the concentration reached 2.2%. A similar effect was observed in our study: higher concentrations of H_2_O_2_ either inhibited seed germination or led to seed death. This raises an important question: are seeds treated with higher concentrations of H_2_O_2_ irreversibly dead, or is their germination merely temporarily suppressed?

### 4.3 Does an elevated concentration of H_2_O_2_ induce cell death in *B.napus*?

Li et al. (2014) investigated the effects of varying H_2_O_2_ concentrations on *Pinus bungeana* seed germination and found that concentrations exceeding 1.2% significantly reduced germination rates compared to the control group, with higher concentrations resulting in progressively lower germination performance. In this study, treatment with 1.2% and 1.3% H_2_O_2_ resulted in germination rates of only 38.89% and 22.22%, respectively. These findings are consistent with those reported by Li et al. (2014). To verify this experimental result, in this study, the seeds that had been treated with H_2_O_2_ for 4 days were subjected to H_2_O treatment again. Notably, following rehydration, the germination rates of seeds treated with 1.2% and 1.3% H_2_O_2_ increased by approximately 30- and 40-fold, respectively. Similarly, the germination rates of seeds treated with 1.4% and 1.5% H_2_O_2_ improved by nearly 50-fold after rehydration, reaching up to 50%. Collectively, these results suggest that high concentrations of H_2_O_2_ inhibit the germination of *B.napus* seeds; however, the inhibitory effect is partially alleviated through rehydration. Therefore, 1.2% and 1.3% H_2_O_2_ cannot be classified as semi-lethal concentrations for *B.napus* seed germination, whereas 1.4% and 1.5% H_2_O_2_ may represent the semi-lethal thresholds for ROS during the growth and development of this species.

Higher concentrations of H_2_O_2_ reduce cell survival rates. For instance, when cells were cultured with hydrogen peroxide alone, their survival rates were 41.61% at a concentration of 100 mM and 7.95% at 200 mM (Mok et al., 2014). Lapshina et al (2016) found that tobacco leaf treatment with 100 mM H_2_O_2_ increased their content of endogenous H_2_O_2_ and changes in the cell structure. Sun et al. (2020) treated strains with H_2_O_2_ at concentrations of 0.25%, 0.50%, 0.75%, 1.00%, 1.50%, 2.00%, 2.50%, and 3.00%. Their findings revealed that when H_2_O_2_ concentrations exceeded 2%, strain biomass approached zero, with 2.5% identified as the lethal concentration for the tested strains. Our study revealed that exposure to 2.2%–3% H_2_O_2_ resulted in germination rates below 10%, with a trend toward near-zero germination as the concentration increased. These results suggest that H_2_O_2_ concentrations exceeding 2.2% are likely lethal to the germination of cabbage-type rapeseed. To further verify these findings, higher concentrations of H_2_O_2_ were applied to healthy plants via spraying. Observations indicated that as H_2_O_2_ concentrations increased, leaves gradually yellowed, with concentrations exceeding 2.2%H_2_O_2_ leading to leaf death. Yang et al (2007) reported that elevated concentrations of H_2_O_2_ resulted in reduced contents of chlorophyll a, chlorophyll b, and total chlorophyll in garlic leaves. These findings, together with the aforementioned observations, indicate that chlorophyll content exhibits a negative correlation with H_2_O_2_ concentration. Furthermore, high levels of ROS—specifically, H_2_O_2_ concentrations exceeding 2.1%—can induce cell death in *B.napus*. Collectively, these results confirming that high levels of H_2_O_2_ concentrations exceeding 2.2% — induce cell death in nasturtium-type rapeseed.

### 4.4 Analysis of changes in the contents of H_2_O_2_, O_2_^-^, SOD, POD and CAT after treatment with different concentrations of H_2_O_2_

Research reported that the levels of ROS generated in cells are tightly controlled by ROS-scavenging enzymes, including SOD, CAT, and POD. These enzymes help maintain ROS at critical concentrations—referred to as the “oxidative window”—enabling them to function as cellular messengers (Bailly et al., 2008). Specifically, O_2_^-^ are rapidly converted to H_2_O_2_ by cellular SOD. Subsequently, the activities of POD and CAT further regulate H_2_O_2_ within the “oxidative window” (Anand et al., 2019). When the concentration of H_2_O_2_ exceeds the critical threshold, it induces the inactivation of relevant antioxidant enzymes. This disruption perturbs the dynamic equilibrium of intracellular ROS (H_2_O_2_ and O_2_^-^), resulting in their substantial accumulation and subsequent triggering of cellular programmed death. After treating wheat (Li et al., 2011), cucumber (Ahmad et al., 2021)seedlings with exogenous H_2_O_2_, it was found that an appropriate concentration of H_2_O_2_ could significantly increase the enzyme activities of SOD, CAT, POD, etc. in the seedlings, reduce the production of H_2_O_2_ and O_2_^-^ in the seedlings. Li et al (2011) reported that pretreatment with 0.05 μM H_2_O_2_ (equivalent to<0.1% H_2_O_2_) enhanced the activities of CAT, SOD, and POD in wheat seedlings. Studies have shown that treating *Tartary buckwheat* seeds with an appropriate concentration of hydrogen peroxide (5–10 mM) can effectively enhance the activities of SOD, CAT, and POD; in contrast, a high concentration of hydrogen peroxide (100 mM) results in reduced activities of these enzymes (Yao et al., 2021). This study found that when the H_2_O_2_ concentration within the appropriate range (0.3% - 0.6%) was applied to *B.napus* seeds, the germination rate, SOD, POD and CAT enzyme activities reached the highest values and were significantly higher than the normal (0.0%) treatment. The higher enzyme activity further reduced the accumulation of H_2_O_2_ and O_2_^-^ in the *B.napus* seed, this result is consistent with the results of NBT and DAB staining. This study further confirmed the previous research viewpoint: lower concentrations of H_2_O_2_ promote the antioxidant enzyme activity of ROS.

However, high concentrations of H_2_O_2_ reduce the antioxidant enzyme activity and have a significant inhibitory effect on plant growth and development (Li et al., 2011; Yao et al., 2021). This study found that as the H_2_O_2_ (0.7%-3.0%)concentration increased, the antioxidant enzyme activity gradually dropped to the lowest level. After 2.2% H_2_O_2_ treatment, the germination rate was 0%, and the enzyme activities of SOD, POD and CAT dropped to the lowest values. There was no significant difference in the enzyme activities among the treatments. This further proved that the H_2_O_2_ concentration (>2.2%) is the lethal concentration threshold for the growth and development of *B.napus*. This result can also reasonably explain that under high concentration H_2_O_2_ treatment, the generation and clearance ability of ROS drop to the lowest level, the seeds die, and the cell permeability of the seeds increases, more exogenous H_2_O_2_ penetrates into the seeds, so the content of H_2_O_2_ in the range of 2.2% - 3% shows a sharp increase trend. This further proves the previous researchers’ viewpoints, high concentrations of H_2_O_2_ reduce the antioxidant enzyme activity and have a significant inhibitory effect on plant growth and development.

## Data Availability

The original contributions presented in the study are included in the article/**Supplementary Material**. Further inquiries can be directed to the corresponding author/s.
