# Supplementary material for: Analysis of the threshold range of ROS concentration in winter rapeseed of the Brassica napus type
Source: Front Plant Sci. 2025 Sep 15;16:1673768. doi: 10.3389/fpls.2025.1673768 (PMC12477161; doi:10.3389/fpls.2025.1673768)
Supplement: Supplementary file 1 [file Table1.docx]

**Appendix table 1 The effects of varying H_2_O_2_ concentrations on the germination rate**

| Varying H_2_O_2_ Concentrations | Seed germination rate (%) | | | |
| --- | --- | --- | --- | --- |
|  | 1d | 2d | 3d | 4d |
| 0.0% | 47.22 ± 1.20 a | 65.00 ± 1.38 f | 80.94 ± 1.91 c | 83.64 ± 1.45 e |
| 0.1% | 38.89 ± 1.17 b | 66.72 ± 1.72 e | 81.64 ± 2.15 c | 83.85 ± 2.01 e |
| 0.2% | 36.72 ± 1.08 c | 69.86 ± 2.05 d | 82.94 ± 1.83 c | 84.33 ± 1.73 d |
| 0.3% | 19.44 ± 0.62 d | 70.30 ± 1.92 d | 83.33 ± 2.03 b | 85.03 ± 1.93 c |
| 0.4% | 13.19 ±0.38 e | 70.47 ± 1.43 c | 83.81 ± 1.72 b | 85.72 ± 2.12 c |
| 0.5% | 12.91 ± 0.93 f | 72.97 ± 1.85 b | 84.17 ± 2.26 a | 89.01 ± 2.04 b |
| 0.6% | 10.58 ± 0.23 g | 74.31 ± 1.65 a | 84.72 ± 1.43 a | 94.67 ± 1.85 a |
| 0.7% | 7.63 ± 0.12 h | 65.28 ± 1.38 f | 75.69 ± 1.92 d | 84.33 ± 1.37 d |
| 0.8% | 6.94 ± 0.21 i | 55.55 ± 1.26 g | 72.08 ± 1.28 e | 81.81 ± 1.69 f |
| 0.9% | 6.33 ± 0.32 i | 49.31 ± 0.76 h | 70.41 ± 1.25 f | 78.78 ± 1.54 g |
| 1.0% | 5.55 ± 0.18 j | 44.44 ± 1.03 i | 59.03 ± 1.33 g | 73.61 ± 1.51 h |
| 1.1% | 2.77 ± 0.06 k | 17.36 ± 0.72 j | 24.31 ± 0.99 h | 60.56 ± 1.74 i |
| 1.2% | 1.39 ± 0.04 l | 13.19 ± 0.06 k | 20.69 ± 1.35 i | 38.89 ± 1.03 j |
| 1.3% | 0.00 ± 0.00 m | 6.25 ± 0.11 l | 10.42 ± 0.62 j | 22.22 ± 1.16 k |
| 1.4% | 0.00 ± 0.00 m | 3.75 ± 0.07 m | 6.81 ± 0.22 k | 10.97 ± 0.83 l |
| 1.5% | 0.00 ± 0.00 m | 1.39 ± 0.03 n | 5.56 ± 0.38 l | 9.03 ± 0.81 l |
| 1.6% | 0.00 ± 0.00 m | 1.25 ± 0.12 n | 5.19 ± 0.45 l | 8.75 ± 0.67 m |
| 1.7% | 0.00 ± 0.00 m | 1.24 ± 0.15 n | 4.56 ± 0.61 m | 6.94 ± 0.49 o |
| 1.8% | 0.00 ± 0.00 m | 1.17 ± 1.13 o | 4.33 ± 0.18 m | 5.50 ± 0.37 p |
| 1.9% | 0.00 ± 0.00 m | 1.11 ± 0.02 o | 4.17 ± 0.36 m | 4.86 ± 0.31 q |
| 2.0% | 0.00 ± 0.00 m | 1.08 ± 0.05 o | 3.47 ± 0.18 n | 4.86 ± 0.27 q |
| 2.1% | 0.00 ± 0.00 m | 1.08 ± 0.06 o | 2.17 ± 0.31 o | 3.86 ± 0.19 r |
| 2.2% | 0.00 ± 0.00 m | 0.00 ± 0.00 p | 0.00 ± 0.00 p | 0.00 ± 0.00 s |
| 2.3% | 0.00 ± 0.00 m | 0.00 ± 0.00 p | 0.00 ± 0.00 p | 0.00 ± 0.00 s |
| 2.4% | 0.00 ± 0.00 m | 0.00 ± 0.00 p | 0.00 ± 0.00 p | 0.00 ± 0.00 s |
| 2.5% | 0.00 ± 0.00 m | 0.00 ± 0.00 p | 0.00 ± 0.00 p | 0.00 ± 0.00 s |
| 2.6% | 0.00 ± 0.00 m | 0.00 ± 0.00 p | 0.00 ± 0.00 p | 0.00 ± 0.00 s |
| 2.7% | 0.00 ± 0.00 m | 0.00 ± 0.00 p | 0.00 ± 0.00 p | 0.00 ± 0.00 s |
| 2.8% | 0.00 ± 0.00 m | 0.00 ± 0.00 p | 0.00 ± 0.00 p | 0.00 ± 0.00 s |
| 2.9% | 0.00 ± 0.00 m | 0.00 ± 0.00 p | 0.00 ± 0.00 p | 0.00 ± 0.00 s |
| 3.0% | 0.00 ± 0.00 m | 0.00 ± 0.00 p | 0.00 ± 0.00 p | 0.00 ± 0.00 s |

**Appendix table 2 Effects of exogenous H_2_O_2_ concentration treatment on various indexes（SOD、POD、CAT、O_2_^-^ andH_2_O_2_） of rapeseed seedlings**

| Concentration of H_2_O_2_ | SOD ［U/g(FW)*h］ | POD ［U/（g·min)］ | CAT ［U/（g·min)］ | O_2_^-^  (noml/g) | H_2_O_2_ (umol/g) |
| --- | --- | --- | --- | --- | --- |
| 0.0% | 61.63±1.79 p | 117.65±1.80 h | 23.01±0.47 h | 56.64±1.50 k | 3.88±0.90 p |
| 0.1% | 212.78±5.86 e | 121.60±2.62 g | 27.45±0.57 g | 76.32±1.20 e | 5.82±0.13 m |
| 0.2% | 230.03±5.92 d | 138.24±3.08 f | 28.01±0.67 g | 78.62±0.60 d | 6.67±0.40 l |
| 0.3% | 390.40±5.36 c | 209.02±2.50 c | 32.13±0.72 d | 73.50±1.10 f | 5.12±0.20 n |
| 0.4% | 427.90±4.04 b | 211.80±3.47 c | 34.32±0.76 c | 71.16±0.75 g | 4.89±0.11 0 |
| 0.5% | 441.10±6.33 a | 216.15±3.52 b | 37.64±0.42 b | 70.78±0.78 h | 4.61±0.21 o |
| 0.6% | 446.01±5.16 a | 227.78±3.59 a | 38.01±0.90 a | 66.36±1.00 j | 4.63±0.20 m |
| 0.7% | 203.40±6.04 f | 205.29±2.24 d | 31.71±0.58 e | 69.32±1.10 h | 6.12±0.01 l |
| 0.8% | 197.67±3.00 g | 181.18±2.03 e | 29.43±0.53 f | 78.37±1.20 d | 7.02±0.02 k |
| 0.9% | 164.76±3.74 h | 121.96±2.44 g | 20.05±0.62 i | 86.68±0.29 c | 7.58±0.12 k |
| 1% | 167.16±2.84 h | 105.00±2.95 i | 18.13±0.28 j | 87.35±1.10 b | 8.20±0.09 j |
| 1.1% | 123.70±3.20 i | 101.06±2.98 i | 17.76±0.55 k | 89.99±0.80 a | 8.67±0.31 j |
| 1.2% | 112.87±2.97 j | 99.63±3.67 j | 16.64±0.61 k | 88.13±1.00 b | 9.90±0.10 i |
| 1.3% | 113.41±2.68 j | 99.29±3.19 j | 10.32±0.64 l | 88.56±1.21 b | 9.88±0.14 i |
| 1.4% | 108.42±4.83 k | 88.66±2.11 k | 9.76±0.70 l | 50.58±1.41 l | 10.70±0.07 h |
| 1.5% | 107.43±3.99 k | 51.43±1.79 l | 8.43±0.44 m | 49.66±0.61 l | 11.14±0.10 h |
| 1.6% | 101.73±4.24 l | 46.91±0.98 m | 7.41±0.35 n | 43.00±1.10 m | 13.48±0.17 g |
| 1.7% | 96.76±2.53 m | 30.95±0.50 n | 7.24±0.25 n | 42.77±0.70 m | 15.34±0.19 f |
| 1.8% | 93.40±0.81 m | 29.00±0.49 n | 7.05±0.32 n | 41.98±0.97 m | 16.33±0.13 e |
| 1.9% | 93.10±2.35 m | 32.10±0.43 n | 6.17±0.53 o | 37.93±0.80 n | 18.58±0.05 d |
| 2.0% | 91.83±2.58 m | 23.92±0.4 o | 5.96±0.28 o | 35.35±1.39 o | 18.92±0.03 d |
| 2.1% | 88.73±1.73 n | 23.65±0.43 o | 3.72±0.53 p | 34.61±0.70 p | 19.29±0.10 d |
| 2.2% | 83.10±2.02 o | 21.91±0.75 0 | 3.42±0.47 p | 34.39±1.30 p | 20.88±0.10 c |
| 2.3% | 80.44±2.070 | 21.09±0.94 0 | 3.13±0.43 p | 33.74±0.80 q | 21.00±0.02 c |
| 2.4% | 80.42±1.90 0 | 19.18±0.50 p | 2.98±0.37 p | 32.99±0.60 q | 21.40±0.12 c |
| 2.5% | 79.30±1.34 0 | 18.82±1.00 q | 2.90±0.34 p | 32.84±0.40 q | 21.85±0.05 c |
| 2.6% | 62.08±1.60 p | 14.29±0.47 q | 2.85±0.15 p | 32.21±1.40 q | 22.70±0.02 b |
| 2.7% | 60.33±1.15 p | 0.00 ±0.00 s | 2.84±0.24 p | 30.84±0.70 i | 23.00±0.05 b |
| 2.8% | 57.60±1.42 q | 0.00 ±0.00 s | 2.76±0.39 p | 28.89±0.70 s | 23.30±0.08 b |
| 2.9% | 35.11±0.87 s | 0.00 ±0.00 s | 2.66±0.31 p | 28.11±0.50 s | 24.17±0.11 a |
| 3% | 30.13±0.85 s | 0.00 ±0.00 s | 2.59±0.32 p | 26.74±1.50 t | 24.96±0.10 a |

**Appendix table 3 After the seeds were pretreatment with H_2_O_2_ (1%-3%) for 4d and then re-watering (4d), effect of H_2_O_2_ and re-watering on seed germination rate of *B.napus* were counted.**

| Handling concentration | Treatment with H_2_O_2_ on the 4th day | Rehydration treatment with H_2_O | | | |
| --- | --- | --- | --- | --- | --- |
|  |  | 1d | 2d | 3d | 4d |
| 1.0% | 73.61±1.51 | 86.67±2.01 | 93.33±2.38 | 93.67±1.83 | 96.67±1.75 |
| 1.1% | 60.56±1.74 | 70.33±1.86 | 83.33±1.18 | 86.67±1.64 | 93.33±2.34 |
| 1.2% | 38.89±1.03 | 70.05±1.38 | 83.33±1.72 | 83.43±1.57 | 86.67±1.63 |
| 1.3% | 22.22±1.16 | 70.00±1.86 | 78.9±1.28 | 80.00±1.05 | 83.33±1.91 |
| 1.4% | 10.97±0.83 | 33.33±1.36 | 43.33±0.98 | 56.67±1.51 | 56.72±1.28 |
| 1.5% | 9.03±0.81 | 30.00±1.07 | 36.67±0.53 | 46.67±1.34 | 50.00±1.82 |
| 1.6% | 8.75±0.67 | 23.33±1.14 | 30.00±1.23 | 36.67±0.23 | 43.33±0.84 |
| 1.7% | 6.94±0.49 | 11.52±0.71 | 23.33±1.08 | 30.00±2.42 | 33.33±1.81 |
| 1.8% | 5.5±0.37 | 10.00±0.42 | 20.00+1.71 | 26.67±1.83 | 30.00±1.28 |
| 1.9% | 4.86±0.31 | 6.67±0.28 | 13.33±0.74 | 20.00±1.15 | 21.21±1.08 |
| 2.0% | 4.86±0.27 | 6.31±0.74 | 10.00±0.82 | 13.33±0.43 | 16.67±1.05 |
| 2.1% | 3.86±0.19 | 5.30±0.18 | 7.46±0.46 | 11.02±1.54 | 12.88±0.67 |
| 2.2% | 0.00±0.00 | 4.93±0.37 | 6.32±0.26 | 9.67±0.31 | 9.86±1.07 |
| 2.3% | 0.00±0.00 | 4.41±0.25 | 5.45±0.67 | 8.96±0.36 | 9.65±1.16 |
| 2.4% | 0.00±0.00 | 4.07±0.24 | 4.87±0.53 | 7.99±0.68 | 8.77±0.73 |
| 2.5% | 0.00±0.00 | 3.83±0.11 | 4.39±0.19 | 6.57±0.24 | 8.01±0.65 |
| 2.6% | 0.00±0.00 | 3.44±0.28 | 4.00±0.81 | 6.09±0.41 | 7.97±0.35 |
| 2.7% | 0.00±0.00 | 2.90±0.14 | 3.68±0.17 | 4.97±0.26 | 5.86±0.33 |
| 2.8% | 0.00±0.00 | 2.61±0.18 | 3.51±0.29 | 3.99±0.35 | 4.99±0.21 |
| 2.9% | 0.00±0.00 | 0.00±0.00 | 0.00±0.00 | 0.00±0.00 | 0.00±0.00 |
| 3.0% | 0.00±0.00 | 0.00±0.00 | 0.00±0.00 | 0.00±0.00 | 0.00±0.00 |
